# TGF-β loaded exosome enhances ischemic wound healing *in vitro* and *in vivo*

**DOI:** 10.7150/thno.57701

**Published:** 2021-04-30

**Authors:** Ao Shi, Jialun Li, Xinyuan Qiu, Michael Sabbah, Soulmaz Boroumand, Tony Chieh-Ting Huang, Chunfeng Zhao, Andre Terzic, Atta Behfar, Steven L Moran

**Affiliations:** 1 Van Cleve Cardiac Regenerative Medicine Program, Center for Regenerative Medicine, Mayo Clinic, Rochester, MN, USA.; 2 Department of Cardiovascular Medicine, Mayo Clinic, Rochester, MN, USA.; 3 Department of Biochemistry and Molecular Biology, Mayo Graduate School of Biomedical Science, Mayo Clinic, Rochester, MN, USA.; 4 Division of Plastic Surgery, Mayo Clinic, Rochester, MN, USA.; 5 Department of Biology and Chemistry, College of Liberal Arts and Sciences, National University of Defense Technology, Changsha, China.; 6 Department of Orthopedic Surgery, Mayo Clinic, Rochester, MN, USA.; 7 Department of Molecular Pharmacology and Experimental Therapeutics, Mayo Clinic, Rochester, MN, USA.; 8 Department of Clinical Genomics, Mayo Clinic, Rochester, MN, USA.; 9 Department of Plastic Surgery, Union Hospital, Huazhong University of Science and Technology, Wuhan, China.

**Keywords:** exosome, wound healing, skin regeneration, translational medicine, cell-free therapy

## Abstract

**Rationale:** With over seven million infections and $25 billion treatment cost, chronic ischemic wounds are one of the most serious complications in the United States. The controlled release of bioactive factor enriched exosome from finbrin gel was a promising strategy to promote wound healing.

**Methods:** To address this unsolved problem, we developed clinical-grade platelets exosome product (PEP), which was incorporate with injectable surgical fibrin sealant (TISSEEL), to promote chronic wound healing and complete skin regeneration. The PEP characterization stimulated cellular activities and *in vivo* rabbit ischemic wound healing capacity of TISSEEL-PEP were performed and analyzed.

**Results:** PEP, enriched with transforming growth factor beta (TGF-β), possessed exosomal characteristics including exosome size, morphology, and typical markers including CD63, CD9, and ALG-2-interacting protein X (Alix). *In vitro*, PEP significantly promoted cell proliferation, migration, tube formation, as well as skin organoid formation. Topical treatment of ischemic wounds with TISSEEL-PEP promoted full-thickness healing with the reacquisition of hair follicles and sebaceous glands. Superior to untreated and TISSEEL-only treated controls, TISSEEL-PEP drove cutaneous healing associated with collagen synthesis and restoration of dermal architecture. Furthermore, PEP promoted epithelial and vascular cell activity enhancing angiogenesis to restore blood flow and mature skin function. Transcriptome deconvolution of TISSEEL-PEP versus TISSEEL-only treated wounds prioritized regenerative pathways encompassing neovascularization, matrix remodeling and tissue growth.

**Conclusion:** This room-temperature stable, lyophilized exosome product is thus capable of delivering a bioactive transforming growth factor beta to drive regenerative events.

## Introduction

Ischemic wounds affect millions of individuals globally and may result in life-threatening amputations and severe morbidities 1-3. Wounds arise from disrupted cellular proliferation, impaired angiogenesis and limited epithelization 4,5. The current treatment options such as wound dressing, application of topical medications and surgery do not restore normal skin anatomy and architecture 6. Cell therapies are believed to improve the sub-optimal outcome from of the traditional treatments. However, they are expensive and require special technical expertise 7,8. This underscores the need to develop simple but effective regenerative alternatives 9. Previous trials of cell-based therapies in wound healing have shown its therapeutic benefits, while the poor survival of transplanted cells, tumorigenicity and immune rejection have been major challenges 10,11.

Meanwhile, extracellular vesicles (EVs) and their exosome subsets are an alternative option for wound healing 12,13. They override the disadvantages presented by cell-based therapies, and mediate wound healing by promoting angiogenesis 14-16, proliferation and migration of cells 17,18 and epithelialization 19. Transferrable through the cell membrane to mediate cell-cell communication, exosomes are highly uniform cell-secreted vesicles ranging from 30-150 nm in diameter, capable of shuttling lipid-encapsulated signaling proteins and nucleotides between cells 16-18.

Platelet-derived agents have long been targets for wound healing. In this study, we assessed the utility of a purified CD63, CD9 and ALG-2-interacting protein X (Alix) positive exosome population (PEP) from activated platelets to promote wound healing. PEP is manufactured and lyophilized under good manufacturing practices as a cell-free regenerative platform to serve as an accessible and cost-effective treatment for wound healing 17,23,24.

Evaluation of PEP has revealed that it accelerates wound healing by releasing bioactive transforming growth factor beta (TGF-β) into the wound bed. Sustained delivery through fibrin sealant (TISSEEL) yielded significant regenerative benefit for full-thickness ischemic wound healing. The results herein provide evidence of the potential ability to preserve TGF-β bioactivity in a lyophilized exosome product applied to accelerate wound healing.

## Methods

### PEP preparation

Clinical grade Purified Exosome Product (Rion LLC, Rochester, MN) vials were acquired from the Advanced Product Incubator biomanufacturing facility at Mayo Clinic (Rochester, USA). Briefly, PEP represents a highly regenerative exosome fraction that is isolated from the conditioned medium of apheresis purified platelets without addition of excipients or activating agents. PEP is obtained through serial filtration, enucleation and centrifugation steps. Final encapsulation step allows lyophilization of the exosome population retaining the integrity of the exosomal lipid bilayer. The derived product is released under Current Good Manufacturing Practice (CGMP) for purity, potency and sterility. Additional process details are provided in the U.S. Patent Pending 20160324A1. A vial of sealed PEP (representing 5×10^12^ exosomes) was dissolved in 1 mL of phosphate buffered saline (PBS, Gibco, USA), and the concentration of solution was defined as 100% (w/v). Before using, the PEP solution was filtered by using a Steriflip®-GP sterile 0.22 μm filter system (Millipore, USA). The 100% PEP solution (5×10^12^ exosomes/ml) was diluted in PBS for characterization or dissolved in culture medium for cell culture.

### Fibroblast exosome isolation

Human dermal fibroblasts (FBs) were maintained in a 37 °C humidified chamber with 5% CO_2_ in DMEM with 10% fetal bovine serum. FBs were cultured until 70% confluency in 100 mm culture dishes. After washing two times with PBS, cells were cultured in DMEM supplemented with exosome depleted FBS (5%). After 72 h, supernatant was collected and centrifuged at 300 × g for 10 min to remove dead cells and debris. Then, fibroblast derived exosomes (FibExo) were isolated by using ultracentrifugation at 120,000 x g at 4 ºC overnight.

### Characterization of PEP (NanoSight and Electron Microscopy)

A drop of PEP on Formvar Carbon-coated nickel grids was stained with 2% uranyl acetate, air-dried and observed under a H-7650 transmission electron microscope (TEM, Hitachi, Tokyo, Japan) to validate their structural integrity. The concentration and size of the vesicle was assessed using a NanoSight NS300 (Malvern Instruments, Malvern, UK). The protein concentration in the vesicles was analyzed using a BCA Protein Assay Kit (Thermo Scientific, USA).

### Western blot analysis

PEP vesicles were lysed in RIPA based lysis buffer and homogenized with an ultrasonic homogenizer (Branson). Protein was quantified using a Pierce BCA Protein Assay kit (Thermo Scientific, USA). Equal amounts of protein were dissolved with SDS-PAGE gel and probed on to Odyssey® Nitrocellulose Membranes (LI-COR Biosciences). Overnight incubation of primary antibodies against CD63 (1:1000, Abcam ab59479), CD9 (1:1000, Cell Signaling 13174s), Alix (1:1000, Cell Signaling 2171s), GAPDH (1:1000, Cell Signaling 2118s), followed by secondary antibody dilusions according to manufacture specification (Invitrogen). Bound antibody was detected using the Odyssey System (LI-COR Biosciences).

### Quantitative real-time polymerase chain reaction (qRT-PCR)

The PEP stimulated gene expressions of human keratinocytes (ABC-TC536S, Accegen, USA) and dermal fibroblasts (C0135C, Gibco, USA) were tested by qRT-PCR. The keratinocytes and fibroblasts were treated with 5% PEP (2.5×10^11^ exosome/ml) for 3 h, 12 h, 24 h while serum free DMEM served as control. Total RNA was isolated from keratinocytes and fibroblasts using TRI Reagent^®^ (Sigma-Aldrich, USA) following the manufacturer's standard protocol. Complementary DNA (cDNA) was reverse-transcribed from equal amounts of RNA (1 μg) by iScript™ cDNA Synthesis Kit (Bio-Rad, USA). All runs were performed using SYBR Green PCR Master Mix (Quantabio, USA) on a C1000 Touch™ Thermal Cycler (Bio-Rad Laboratories, USA) for the following genes: smad2, ras, mkk3, erk1, periostin, p38, rhoa, and tak1. Glyceraldehyde-3-phosphate dehydrogenase (GAPDH) was selected as an internal control. To control for pipetting errors, each cDNA sample was run in duplicate. The primers used in the amplification were listed in Table 1. Data from target genes was normalized to GAPDH and then calculated using the 2^-ΔCt^ method.

### Macrophage polarization assay

Fresh bone marrow was isolated from rabbit long bones and cultured in RPMI 1640 (Invitrogen, USA) supplemented with 10% heat inactivated fetal bovine serum, 1× Penicillin-Streptomycin solution (ThermoFisher, USA) 10 ng/ ml M-CSF (Sigma, USA) and 50 pM ß-mercaptoethanol (Gibco, USA). A week later, attached cells were identified as macrophages and were treated accordingly. The cells were classified into several groups. The LPS and LPS+PEP groups were pre-treated with 50 ng/ml LPS for 2 h before adding serum free RPMI medium or 5% PEP (2.5×10^11^ exosome/ml) supplemented RPMI medium. After 24 h, cells were collected and run for qRT-PCR as mentioned above, while cells cultured in serum rich RPMI medium served as control. Cells were then probed for the following genes: nos-2, tnf-a, IL-6, IL-1b, IL-10, CD206.

### Enzyme-linked immunosorbent assay (ELISA)

TGF-β, VEGF, PDGF, Collagen I and Collagen III concentration was measured separately by ELISA (R&D system, Minneapolis, MN, USA; Thermo Scientific, USA). The absorbance at 450 nm was measured using a 96-well microplate spectrophotometer (FLUOstar Omega, BMG LABTECH, USA).

### Cell proliferation assay

IncuCyte® 96-well proliferation assay (Essen BioScience, MI, USA) was performed according to the manufacturer's protocol. Human keratinocytes and human dermal fibroblasts were seeded in a 96-well microplate. The plate was placed in IncuCyte and images were automatically acquired 48 h post incubation. We used the Cell Proliferation Module (Essen BioScience) to quantify the proliferation rate. Eight biological replicates were included for each condition.

To examine the effects of TGF-β and PDGF, PEP exosomes were pre-incubated with a 10 ug/mL pan-TGFβ (R&D Systems, MN, USA) or PDGF (Millipore, MA, USA) neutralizing antibody that blocks the TGFβ or PDGF interaction with its receptor. Moreover, we added 10 nM PDGFR inhibitor (CP 673451, R&D Systems, MN, USA) treatment as another control group that selectively blocked PDGF signaling.

### HUVEC angiogenesis assay

IncuCyte® 96-well angiogenesis assay (Essen BioScience, MI, USA) was performed according to the manufacturer's protocol. Briefly, lentivirus infected green fluorescent protein (GFP) expressing HUVECs (Essen BioScience) were co-cultured with normal human dermal fibroblasts (Essen BioScience) in a 96-well microplate. The plate was placed in IncuCyte and images were automatically acquired in both phase and fluorescence every 6 h for 10 d. HUVECs were cultured in Angiogenesis Prime Kit Optimized Assay Medium (Essen BioScience) supplemented with designated treatments and kept throughout the experiment. We used the Angiogenesis Analysis Module (Essen BioScience) to quantify tube length and branch points. Eight biological replicates were included for each condition.

### Cell migration assay

Primary rabbit dermal fibroblasts were isolated from healthy rabbit (around 6 months old with a body weight of 2.0-3.5 kg) ear skin and maintained in DMEM with 10% fetal bovine serum. Primary human keratinocytes (hKC) were purchased from Gibco (C0055C, Thermofisher Scientific, USA) and maintained in EpiLife medium supplemented with Human Keratinocyte Growth Supplement Kit (Thermofisher Scientific). The cells were serum deprived for 24 h followed by 2 h treatment of 10 µg/ml mitomycin C (Sigma, USA) before starting the experiments. Cell migration was analyzed by scratch wound assay, which was measured by IncuCyte S3 live cell imaging system (Essen Bioscience). rFBs or hKCs were seeded in the 96-well plate (Corning). Cells proliferated in standard tissue culture condition until confluent, followed by scratching on cell monolayer using Wound Maker (Essen Bioscience). After two phosphate-buffered saline washes and addition of 10% PEP with blank medium (DMEM for rFB and EpiLife medium for hKC), the plate was placed into IncuCyte for timed imaging.

### Skin tissue organoid assay

Skin tissue organoid assay (ThermoFisher Scientific, USA) was performed according to the manufacturer's protocol. Briefly, Human adult epidermal cells (C0055C, Gibco, MT, USA) were seeded at 750,000 cells/cm^2^ in pre-coated cell culture inserts and cultured with 50uL EpiLife growth medium with supplements (Gibco, MT, USA). After 2 d of incubation at 37 °C and 5% CO_2_, we repositioned the inserts at the desired hanging height in the 24-well plate and changed medium while the upper compartment, inside of the cell culture insert, was left empty. Skin tissue inserts proliferated for 28 d post-seeding and were then fixed using an overnight incubation in 4% paraformaldehyde at 4 °C. Inserts were paraffin embedded and sectioned followed by processing for hematoxylin and eosin (H&E) staining. Tissue sections were photographed using Olympus BH-2 microscope (Olympus Life Science, PA, USA) at 400× magnification to examine the stratification of cells.

### PEP biogel preparation and SEM

To prepare PEP with TISSEEL, a 2 mL standard kit (Baxter, IL, USA) was utilized. PEP was dissolved in the fibrinolysis inhibitor solution (human fibrinogen, aprotinin, human albumin, L-histidine, niacinamide, natriumcitratdihydrate, polysorbate, water) in the TISSEEL fibrin glue preparation kit. The solution was then prepared following standard TISSEEL preparation protocol per manufacturer specifications and mixed with thrombin solution (500 IE/ml)/CaCl_2_ (40 µmol/ml) for local administration.

For SEM, samples were fixed in Trumps fixative at 4 °C overnight, washed with PBS and then in water, followed by dehydration. They were then imaged in a Hitachi S-4700 cold field emission scanning electron microscope.

### *In vivo* wound healing animal model

Twelve Female New Zealand white rabbits (*Oryctolagus cuniculus*) (around 6 months old with a body weights of 2.0-3.5 kg) were used for this study. Rabbits were assigned into each treatment group in sequence. Ischemia was induced in both ears by ligating 2 of the 3 vascular bundles of the ear. A circular full-thickness skin defect measuring 2 cm in diameter was created on each ear. Ear ischemia after surgery was confirmed using indocyanine green angiography with the SPY Elite florescence imaging system (Stryker, MI, USA). Rabbits were then randomly assigned into 3 groups. In each group, one ear was left as untreated while another side was treated with 0.6 mL TISSEEL, 0.6 mL 20% PEP or 0.6 mL TISSEEL-PEP (20%) combination. Wound healing was observed and recorded every day for the first week and weekly thereafter until rabbits were sacrificed on the 4th week. All animal procedures were approved by Institutional Animal Care and Use Committee of Mayo Clinic (A00004139-18).

### Clinical assessment of wound closure

Wound healing was graded from a clinical perspective. All wounds were assessed weekly by board-certified plastic surgeons at Mayo Clinic and graded according to the Wagner Ulcer Grade Classification System.

### Measurement of wound hydration level and oil level

Skin hydration and oil level were objectively evaluated using a digital skin moisture/oil detector (Zinnor, Korea). All measurements were taken under standard climate conditions (temperature, 25 ± 1 °C; relative humidity, 50 ± 5 percent).

### Biomechanical Test

The specimens of the full thickness wounds were cut into 2 mm wide strips. The specimens were then mounted to a tensile testing device using super glue (Gorilla, OH, USA). The glue was applied to the bare skin on the ends to prevent slipping from occurring at the site where the specimens were clamped with grips. The grips had a serrated internal surface to reduce slipping. A custom tensile testing device, with a Transducer Techniques 25 lb load cell (MLP-25, CA, US), was used to perform cyclic tensile testing of the specimens. Specimens were tested at a constant strain rate of 0.1 mm/s, with a peak displacement of 1 mm for 20 cycles. A preload of 1 N was applied before starting each test. After the 20^th^ cycle, the specimens were tested to failure with a strain rate of 0.1 mm/s. The motor control and data acquisition were handled via a custom NI LabVIEW 2018 application (National Instruments, TX, US), with the load and displacement data sampled at a rate of 50 Hz.

### Histology

Rabbits were sacrificed by 4 weeks post-surgery. Ear skin from the original site of injury was removed and fixed in 10% buffered formalin overnight at 4 °C, then rinsed 24 h at 4 °C in PBS containing 30% sucrose and 0.1% sodium aside, with PBS changes after approximately 12 h to eliminate any formalin remnants. Specimens were then embedded in paraffin wax (Thermo Fisher Scientific, DE, USA) by a tissue processor (Excelsior™ AS Tissue Processor, NY, USA). They were then sliced into longitudinal sections (5 μm) and prepared onto slides (Superfrost® plus, Menzel-Gläser, Germany). After embedding, the specimens were transversally sectioned in 5 µM for further use. Hematoxylin and eosin (H&E) staining and Masson's trichrome staining were performed according to standard procedures.

### Immunohistochemistry IHC analysis (TGF-β, Col I & III, α-Smooth Muscle Actin, CD31)

Skin sections underwent deparaffinization and were permeabilized with 0.5% Triton X-100 in PBS for 5 min. They were next incubated with blocking buffer (5% normal donkey serum, 0.2% Triton-X in PBS) prior to primary antibody incubation at 4 °C overnight with the following antibodies diluted in blocking buffer: anti-TGF-β(1:400, MAB240-SP, R&D system, MN, US), anti-Collagen I (1:200, ab24821, Abcam, Cambridge, UK), and anti-Collagen III (1:400, ab6310, Abcam). After three 30-min washes with PBS + 0.05% Triton-X, samples were stained for 1 h at room temperature with fluorescent secondary antibodies (ThermoFisher Scientific, MA, USA) followed by two washes with PBS. The slides were mounted with Antifade Mounting Medium with DAPI (h-1200, Vector laboratories, CA, USA) and viewed under a spinning-disc confocal microscope (Carl Zeiss).

### 3D-EM reconstruction of wound tissue

Full thickness wound samples were fixed, stained and prepared for serial block-face microscopy using a protocol adapted from that published by Hua *et al*. 25 Briefly, tissue samples were fixed by immersion in 2% glutaraldehyde + 2% paraformaldehyde in 0.15 M cacodylate buffer containing 2 mM calcium chloride until further processing (minimum of 24 h). Fixed samples were washed in 0.15 M cacodylate buffer and incubated at room temperature in 2% osmium tetroxide in 0.15 M cacodylate for 1.5 h. Without rinsing, samples were incubated in 2.5% potassium ferricyanide + 2% osmium tetroxide in 0.15 M cacodylate for another 1.5 h at room temperature. Following a rinse in deionized water (dH_2_O), samples were incubated in 1% thiocarbohydrazide in H_2_O for 45 min at 50 °C. After another rinse in dH_2_O, samples were incubated sequentially in 2% osmium tetroxide in dH_2_O for 1.5 h at room temperature, 1% aqueous uranyl acetate overnight at 4 °C, and 7% lead aspartate solution 1 h at 50 °C, with several rinses in dH_2_O between each reagent. Following dehydration through a series of ethanol and acetone, samples were eventually embedded in polyepoxide resin Durcapan (EMS, Hatfield, PA) and polymerized in a 60 °C oven for a minimum of 24 h. To prepare embedded samples for placement into the SEM and subsequent imaging, 1 mm^3^ pieces were roughly trimmed of any excess resin and mounted to 8 mm aluminum stubs using silver epoxy Epo-Tek (EMS, Hatfield, PA). The mounted sample was then carefully trimmed to a 0.5 mm × 0.5 mm x 1 mm tall tower using a diamond trimming knife (Diatome trimtool 45, EMS, Hatfield, PA). Trimmed sample and entire stub were coated with gold palladium to assist in charge dissipation. The coated sample was then inserted into a VolumeScope serial block-face SEM (Thermo Fisher, Waltham, MA) and allowed to acclimate to high vacuum for 12 h prior to the start of imaging. High resolution block-face images were obtained in a low vacuum environment using a beam energy of 3.0 kV with a current of 100 pA and a scanning dwell time of 2 µs and a 10 nm pixel size. A stack of approximately 500 block-face images were obtained while cutting the block at 50 nm increments. The image stack was then aligned and filtered using Amira software (Thermo Fisher, MA, US) with further analysis performed using Reconstruct 26.

### Drug release assay

Here, 0.3 mL TISSEEL and TISSEEL-PEP biogels were prepared and incubated separately at 37 °C in 2 mL PBS. The concentration of the vesicles in the supernatant at day 1, 3, 7 and 14 were measured using a NanoSight NS300 (Malvern Instruments, Malvern, UK).

### RNA-Seq and data analysis

Wound tissue was harvested 28 days post operation. RNA was extracted using TRIzol Plus RNA Purification kit (Thermofisher Scientific, USA) according to the protocol. RNA library preparations and sequencing reactions were conducted at GENEWIZ, LLC. (South Plainfield, NJ, USA). Data analysis was performed following the standard mRNA analysis pipeline. Expression levels of mRNAs were computed as Normalized Count number for Possion Distribution based statistical analysis 27. Significantly changed genes (|log_2_FC|>0.5 and p <0.05) with relatively high expression levels (Normalized read counts>100) were used for heatmap visualization and further analysis. Gene Ontology (GO) and pathway enrichment analysis were performed by *clusterProfiler* (V3.12.0) 28 and heatmap visualization was conducted by *pheatmap* (V1.0.12) 29.

### Statistical analysis

Quantitative results were expressed as the mean value ± standard error. Statistical analysis was performed using two-way ANOVA and 2-tailed unpaired Student's t test (SPSS software 13.0, USA; GraphPad Prism 8.0, USA). A value of p < 0.05 was considered statistically significant.

## Results

### Extracellular vesicles from PEP exhibit exosomal traits

**Figure 1:**
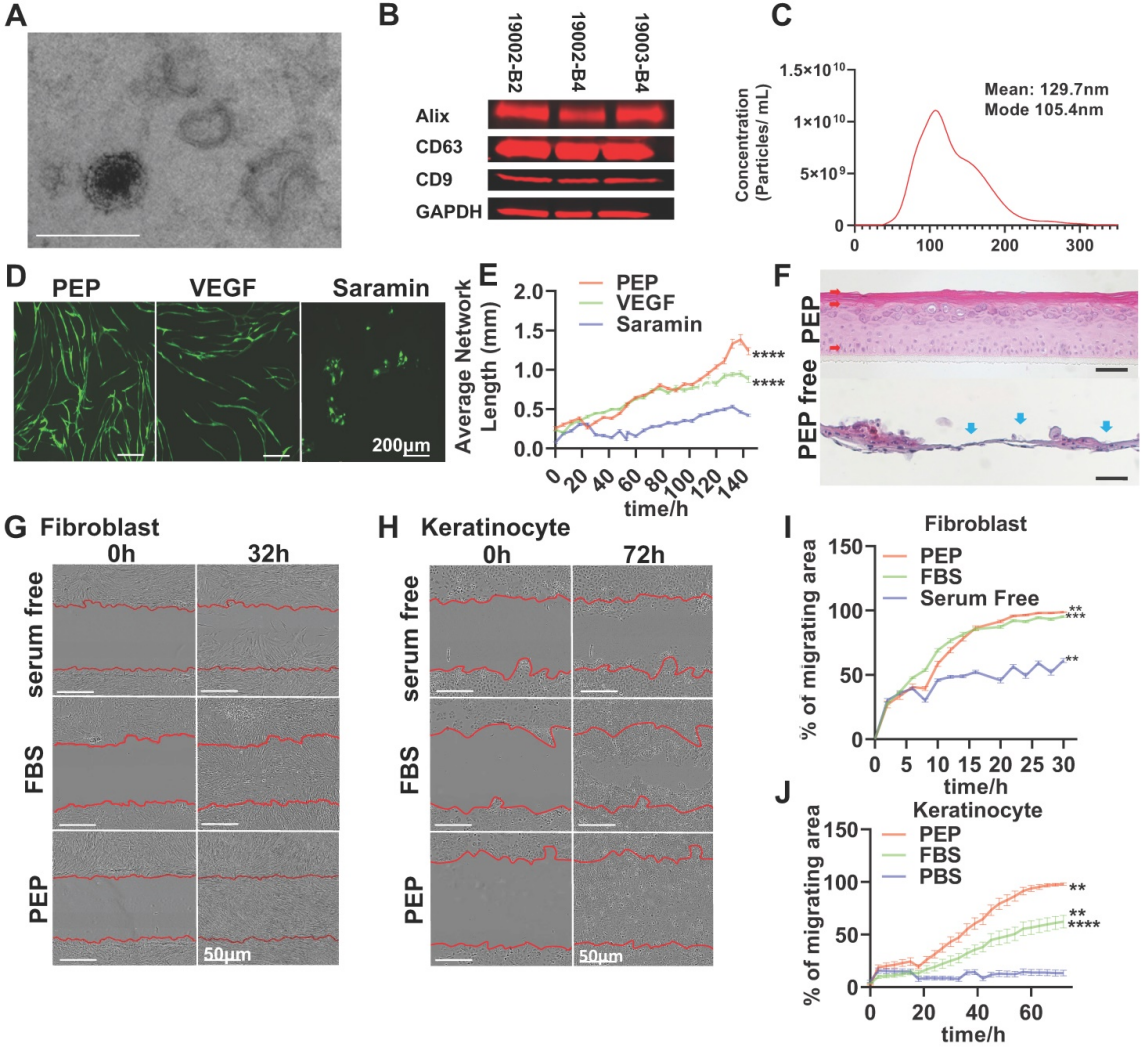
PEP displays exosomal characteristics and drives *in vitro* activities of skin progenitors. (A): Representative transmission electron microscope image for PEP exosomes. (Scale; 1 bar represents 200 nm). (B): Western blot analysis for the expression of CD63, CD9 and Alix on PEP exosomes from three separate current good manufacturing practices lots. Total protein staining was used as a loading control validated by GAPDH probing. (C): Size distribution of PEP exosomes obtained using NTA (NanoSight). The exosomes were averagely 129.4 nm wide. (D): *In vitro* angiogenesis assay using co-culture of human dermal fibroblast (hFB) with GFP-tagged human umbilical vascular endothelial cells (HUVEC) in presence of VEGF, PEP or Suramin. (E): Quantification of average network length in 6 hours increments over a 6 day period. (F): Organoid differentiation assay of human keratinocytes treated PEP or serum free media at day 24. H&E staining was performed to all the groups. Red arrow: Organized differentiated keratinocyte with multiple epidermis-like layers. Blue arrow: unorganized keratinocytes differentiation. (G): Scratch assay evaluating the migration of primary rabbit dermal fibroblast treated with FBS, PEP or serum free media. Representative pictures of wound closure for FBS vs. PEP vs. serum free media at 0 h and 32 h. (H): Scratch assay testing the migration of human keratinocytes treated with FBS, PEP or PBS. Representative pictures of wound closure for FBS vs. PEP vs. PBS at 0hr and 72 h. (I): Graph showing quantification of fibroblast wound closure, performed every 2 h over a 32 h period. (J): Graph showing quantification of keratinocytes wound closure, performed every 3 h over a 72 h period. Scale bar in A&D: 200 µm, Scale bar in B&C: 50 µm. N= 8 in D-J, 2-tailed paired Student's t test for each group compared with control group. ***p < 0.001, ****p < 0.0001.

To ensure uniformity in current good manufacturing practices regenerative exosomes, the PEP vesicles were assessed for morphology, exosomal markers and size (Figure 1A-C). Transmission electron microscopy (TEM) documented an intact double-membrane nanostructure of PEP vesicles (Figure 1A, Figure S1A). Furthermore, the PEP expressed CD63, CD9 and Alix exosomal markers (Figure 1B), consistent with additional release criteria. The PEP has a calculated concentration of protein approximately 60 μg/μL and its endotoxin level was less than 0.100. It's moisture concentration, sterility and biocapability towards cell proliferation were consistent from batch to batch (Figure S1B,C) 30,31. The hydrodynamic diameter of PEP had a mean value of 129.7 nm (Figure 1C). This multi-parametric quality control evaluation helped validate the uniform exosome content of PEP.

Next, PEP stimulation of skin cell healing was evaluated *in vitro* (Figure 1D-J) through effects on neovascularization, cell proliferation and migration. Human umbilical vascular endothelial cells (HUVECs) were cultured with PEP, vascular endothelial growth factor (VEGF) or suramin (a VEGF inhibitor) on a fibroblast monolayer culture. PEP was noted to stimulate angiogenesis in HUVECs more effectively than VEGF, as shown by a marked increase in average network lengths and branch points of endothelial tube formation (Figure 1D, E, Figure S2). Human keratinocytes (hKC) cultured in 3D with PEP showed differentiation of hKC in an air-liquid interface culture and regenerated a normal epidermal architecture within 21 days (Figure 1F). PEP furthermore promoted the proliferation and migration of primary rabbit dermal fibroblasts (rFB) and hKC as documented in proliferation assay (Figure S3) and wound scratch assay (Figure 1G-J).

### PEP TGF-β donation activates downstream pathways

**Figure 2:**
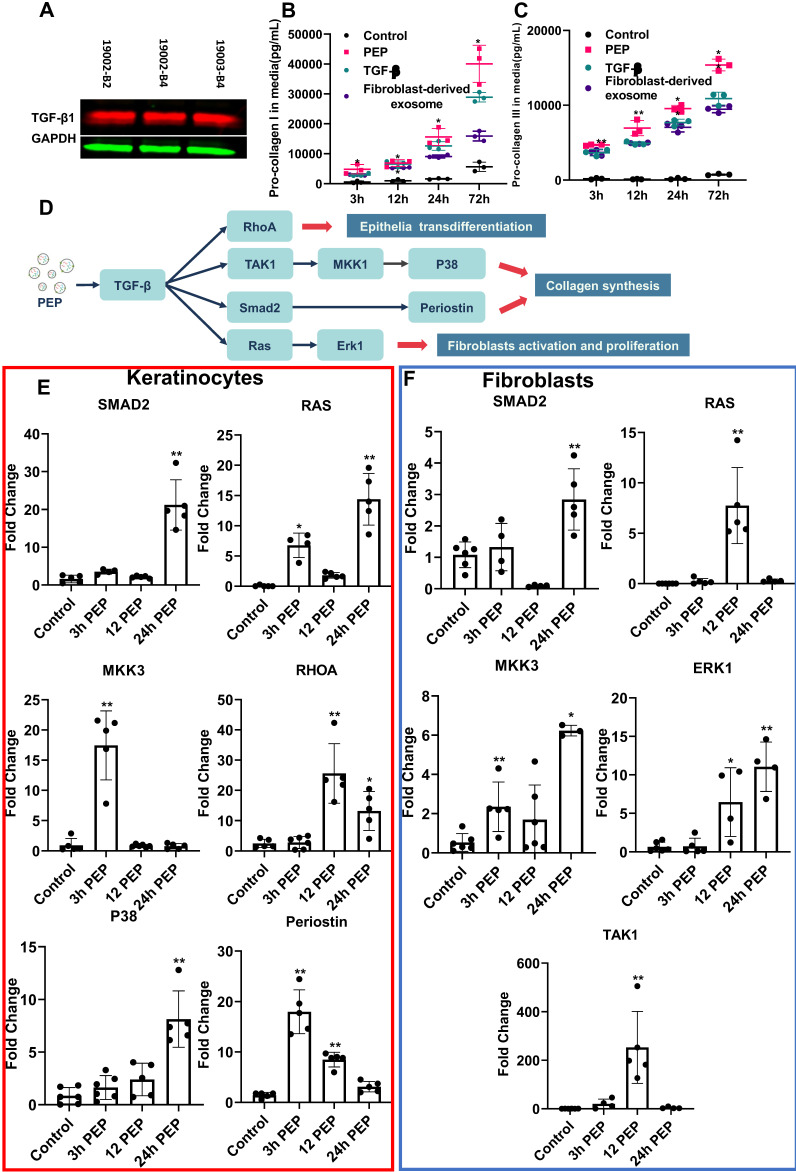
PEP donates bioactive TGF-β activating proliferation in dermal progenitors *in vitro*. (A): Representative western blot of TGF-β1in PEP exosomes. GAPDH was used as a loading control. Pro-collagen I (B) and III (C) synthesis in TGF-β (5 ng/ml), fibroblast-derived exosomes or PEP treated fibroblasts. (D): Schematic illustration of the mechanism of PEP-induced wound healing *in vitro*. (E): Quantification of SMAD2, RAS, MKK3, RHOA, P38, and Periostin expression in PEP treated keratinocytes. (F): Quantification of SMAD2, RAS, MKK3, ERK1, and TAK1 expression in PEP treated fibroblasts. A 2-tailed unpaired Student's t-test was used for each group compared to the untreated control group. *p < 0.05, **p < 0.01.

Further studies of growth factors from PEP pin-pointed encapsulated TGF-β (Figure 2A, Figure S4), as a driver for wound healing events 32,33. Similar to TGF-β stimulation and fibroblast derived-exosome treatments, treatment of human fibroblasts (hFB) with PEP significantly augmented collagen I and III secretions versus TGF-β inhibition and negative control (Figure 2B, 2C). To further study TGF-β activity (mapped in Figure 2D), downstream targets were probed in both hFB and hKC. Compared to control, PEP-treated hKC upregulated TGF-β targets including SMAD2, RAS, Mitogen-activated protein kinase kinase 3 (MKK3), RAS homolog family member A (RHOA), P38 and Periostin, facilitating epithelial transdifferentiation (Figure 2E) 32,34-36. Upregulating expression of the above genes was also observed in PEP-treated hFB (Figure 2F). The increased expression of SMAD2, RAS, MKK3, Extracellular signal-regulated kinases 1 (ERK1) and Transforming growth factor beta-activated kinase 1 (TAK1) also suggested PEP donated TGF-β to stimulate fibroblast wound healing activities 34-36. To confirm the important role of PEP mediated wound healing events *in vitro*, we selectively blocked TGF-β on PEP by pre-incubating PEP with pan-TGF-β-neutralizing antibody before evaluating its biopotency *in vitro*. The TGF-β-neutralized exosomes induced a significant suppression in fibroblast and keratinocyte proliferation, fibroblast migration and collagen synthesis, as well as HUVEC tube formation (Figure S3, S5).

### TISSEEL-PEP biogel promotes ischemic wound healing *in vivo*

**Figure 3:**
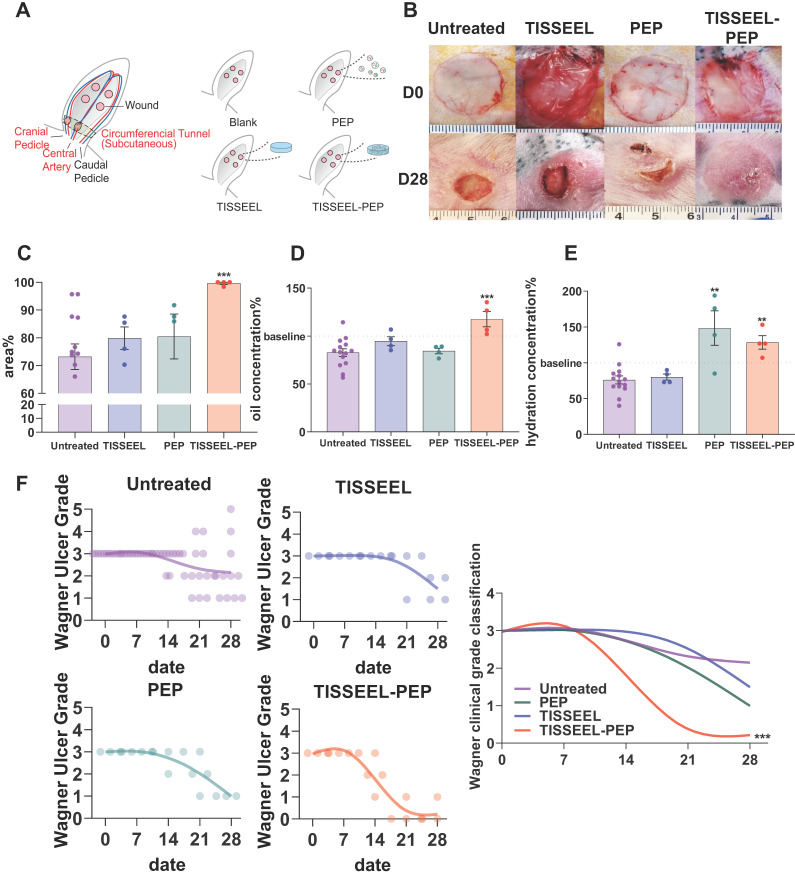
TISSEEL-PEP biogel promotes ischemic wound healing *in vivo*. (A): Schematic of study design. Ligation of arteries produced an ischemic wound environment. Animals were divided into 4 groups: untreated, treated with PEP alone, TISSEL alone or the TISSEEL-PEP biogel. (B): Photographs of representative wounds from each of 4 groups. (C): Bar graphs showing quantification of wound healing. Each bar measures average wound size for each group as a percentage of original wound on day 28. (D), (E): Hydration and oil levels of skin tissue four weeks post injury. The skin hydration and oil level of different treated groups were measured and compared with untreated group. Normal skin served as baseline. (F): Clinical assessment of wound closure (N=12 in untreated group, N=4 in TISSEEL, PEP and TISSEEL-PEP group). All groups were assessed by certificated physician for Wagner Ulcer Classification each week post injury. Each individual data was plotted in the graph with a smoothing spline curve created. A 2-tailed unpaired Student's t-test was used for each group compared to the untreated control group in Figure C-D. 2-way ANOVA for each group compared with untreated control group in Figure F. *p < 0.05, **p < 0.01, ***p < 0.001.

To assess whether PEP can stimulate skin regeneration, a sustained release vehicle was required as exosomes have a high potential for cell absorption and therefor a very short *in vivo* half-life. Accordingly, the clinically established surgical wound sealant (TISSEEL) was utilized as the vehicle for PEP. Wound healing in an ischemic rabbit (*Oryctolagus cuniculus*) ear model was used to evaluate the efficacy of a PEP-biopotentiated TISSEEL biogel (TISSEEL-PEP; Figure S6A) compared with control groups including untreated, TISSEEL-only and PEP-only treated animals (Figure 3A). At day 28, persistent wounds were found in all groups except for the TISSEEL-PEP group (Figure 3B and Figure S6B). The TISSEEL-only group served as vehicle control and PEP-only served to assess rapid exosome release versus the sustained release achieved with TISSEEL-PEP (Figure S7). TISSEEL-PEP induced the fastest wound closure rate (Figure 3B, C, Figure S6B, Figure S8) with evidence of early hair regrowth. Skin hydration and oil levels are important markers of healed skin; both were significantly higher in TISSEEL-PEP treated animals (Figure 3D, 3E), suggesting a restoration of cutaneous homeostasis 37. Results were further supported by Wagner Ulcer classification analysis showing TISSEEL-PEP biogel expedited wound healing (Figure 3F). Collectively, these findings suggest that TISSEEL-PEP facilitates ischemic wound healing.

### PEP contributes to wound tissue reorganization

**Figure 4:**
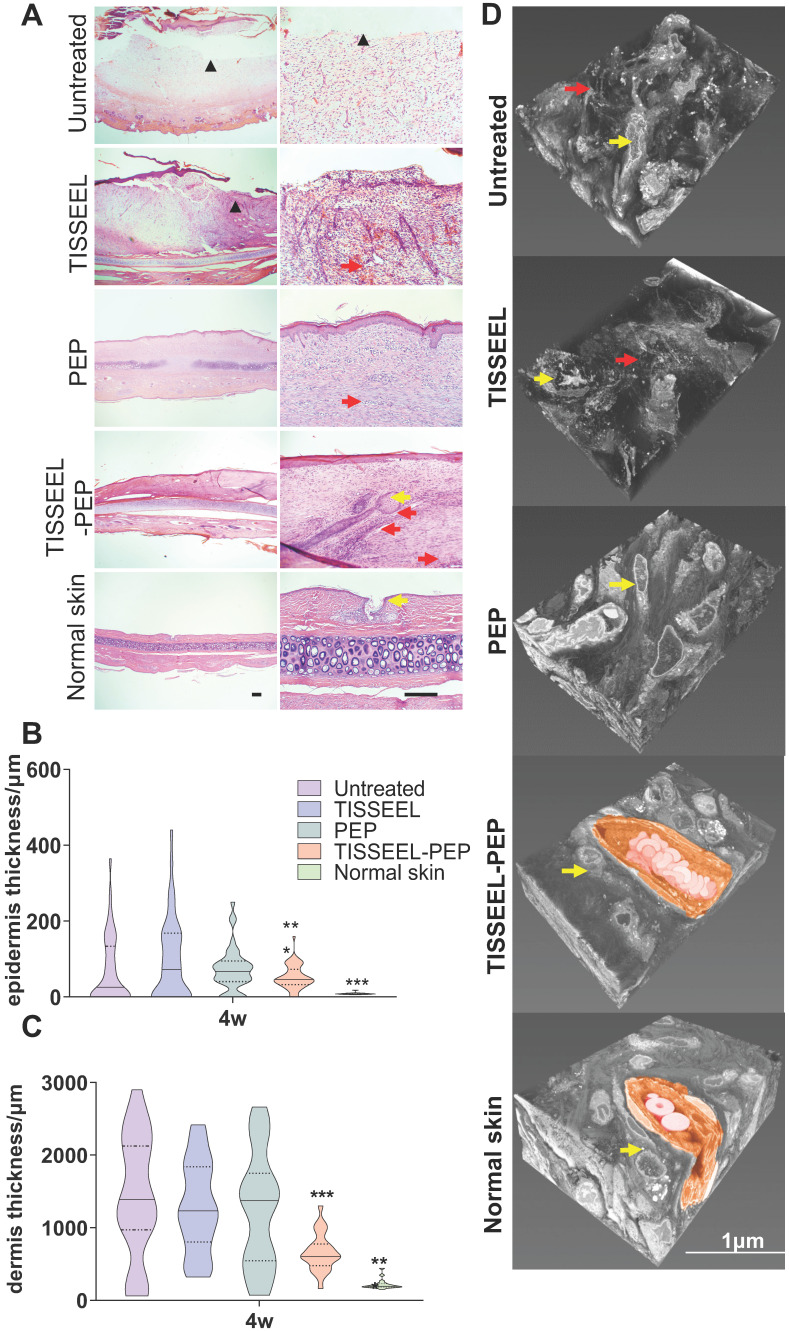
PEP mediated structural reorganization of wound tissue. (A): H&E staining analysis performed on Untreated, TISSEEL-only control, PEP, TISSEEL-PEP and normal skin. Top row and bottom row are representative images from each group. Black scale bar in normal skin column represents 100 µm. ▲: unhealed area. Yellow arrow: hair follicle. Red arrow: new blood vessel. (B), (C): Quantification of epidermal (B) and dermal (C) layer thickness 28 d after treatment performed in 10 slides and 5 different locations per slide representatively. N=4. (D): Representative 3D-EM reconstruction of wound tissue (n=3). Yellow arrow: fibroblast. Red arrow: disorganized collagen deposition. Coloured Region: new capillary with red blood cell. Reference bar in normal skin is 1 µm. A 2-tailed unpaired Student's t-test was used for each group compared to the untreated control group. *p < 0.05, **p < 0.01, ***p < 0.001.

Four-week follow up revealed that TISSEEL-PEP treatment restored normal dermal and vascular architecture, comparable to normal skin (Figure 4A, and Figure S9A, B). The cartilage of 1/3 of the untreated animals remained exposed, with minimal collagen deposition. However, TISSEEL-PEP treatment induced growth of hair follicles and development of sebaceous glands at the wound site, absent in the other groups. In addition, TISSEEL-PEP treatment inhibited scaring of healed wound sites. The transverse sections for the wound tissues revealed normal anatomy of epidermal and dermal structures (Figure 4A-C). PEP stimulates migration and differentiation of keratinocytes, thus accelerating re-epithelization and inhibiting scar formation of wound sites 38. Three-dimensional electron microscopy (3D-EM) of full wound tissue thickness (Figure 4D, Movie S1) revealed that compared to control with disorganized tissues with low collagen fiber content, the collagen fibers of the PEP-only group were well aligned but typical of a new scar. In contrast, TISSEEL-PEP group exhibited a basket-weave collagen structure, which is similar to a mature extracellular matrix in normal skin (Figure 4D).

### PEP biogel drives collagen organization to promote wound healing

**Figure 5:**
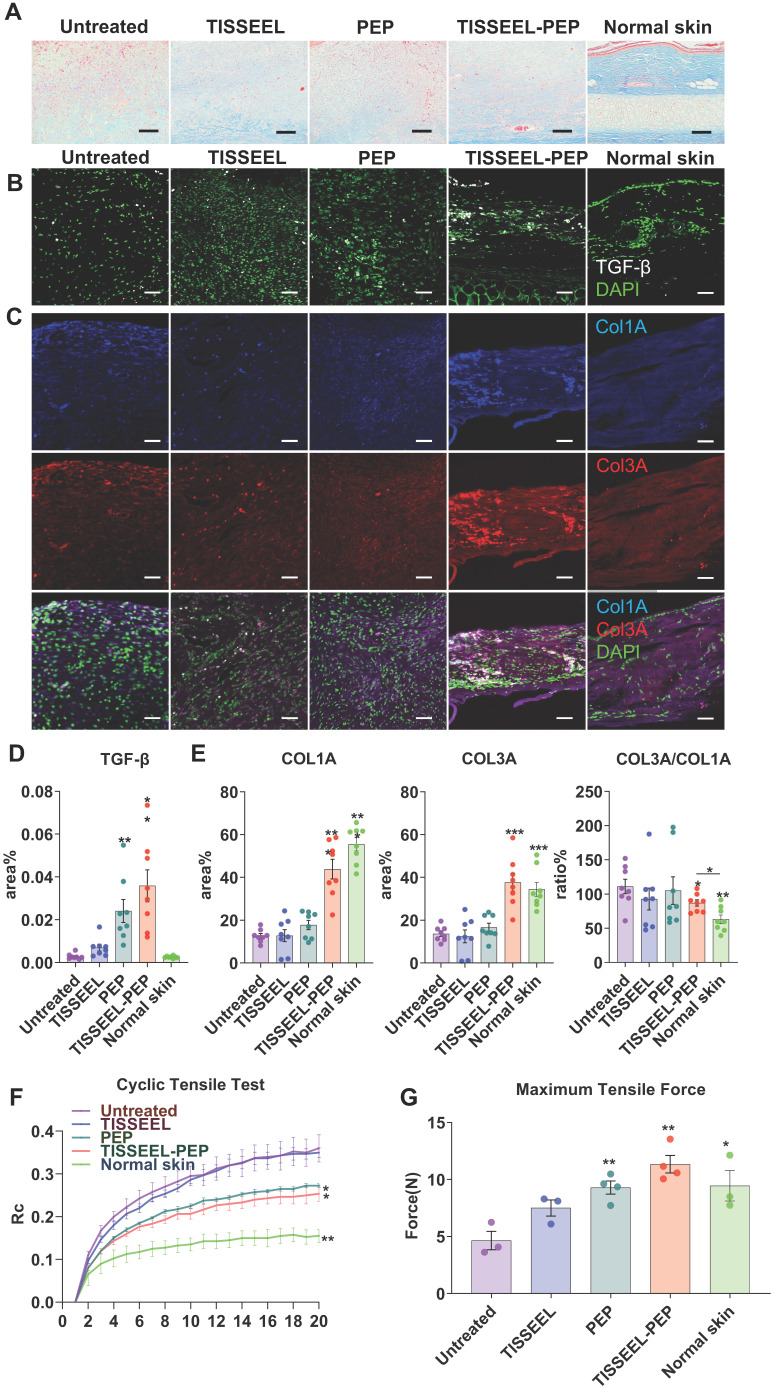
PEP biogel activated TGF-β signaling and promoted collagen organization. (A): Masson Trichrome staining analysis of different groups of wound tissue, as well as normal rabbit ear skin. Skin tissue was obtained at day 28 post-surgery. (B): TGF-β immunofluorescence staining of wounds from different groups. (C): Col1A and Col3A immunofluorescence staining of wounds from different groups. (D), (E): Immunofluorescence quantification for TGF-β, COL1A, COL3A and COL3A to COL1A ratio, N=8. (F): Cyclic tensile test for all the groups. Untreated and TISSEL treated only groups were stiffer and less like normal skin. Rc: reaction force variation. N=4. (G): Maximum tensile test of all the groups. The TISSEEL-PEP group resisted the highest tensile forces, N=4. Scale bar in Figure A: 100 µm. Scale bar in B&C: 200 µm. 2-way ANOVA for each group compared with control group in Figures D and E. 2-tailed unpaired Student's t-test for each group compared with untreated control group in Figure B,C&G. *p < 0.05, **p < 0.01, ***p < 0.001.

Given the distinct collagen content organization among the treatment groups (Figure 4D, 5A, and Figure S9D), we further explored the association between collagen organization and TGF-β expression *in vivo* (Figure 5A-C) 36. Consistent with augmented cell migration (Figure 1G-J) and induction of gene expression downstream of TGF-β (Figure 2E-F) *in vitro*, TISSEEL-PEP stimulated higher tissue expression of TGF-β (Figure 5B,D) 39, which induced synthesis of collagen type I (COL1A) and collagen type III (COL3A) (Figure 5A,C,E) 40. The ratio of COL3A/COL1A was highest in the TISSEEL-PEP group, relative to the normal skin group. This suggests that wound healing with less scaring is mediated by COL3A 41,42. In contrast, apart from scaring, the control group displayed slow wound healing and abnormal collagen architecture (Figure 3B, Figure S6B, Figure S9).

Based on the effect of PEP on collagen concentration and organization, we hypothesized that collagen content affects the skins' biomechanical properties. Cyclic stretching revealed that the skin properties of rats in the PEP-only and TISSEEL-PEP tissue were comparable to that of uninjured skin (Figure 5F). In contrast, the skin elasticity was poorest for the untreated and TISSEEL-only groups. The tensile strength of skin following of TISSEEL-PEP treatment was similar to that of uninjured skin (Figure 5G). These findings suggest that TISSEEL-PEP treatment can induce regeneration of skin tissues, restoring its previous biomechanical properties.

### TISSEEL-PEP treatment triggers transcriptional remodeling underlying pro-wound healing events

**Figure 6:**
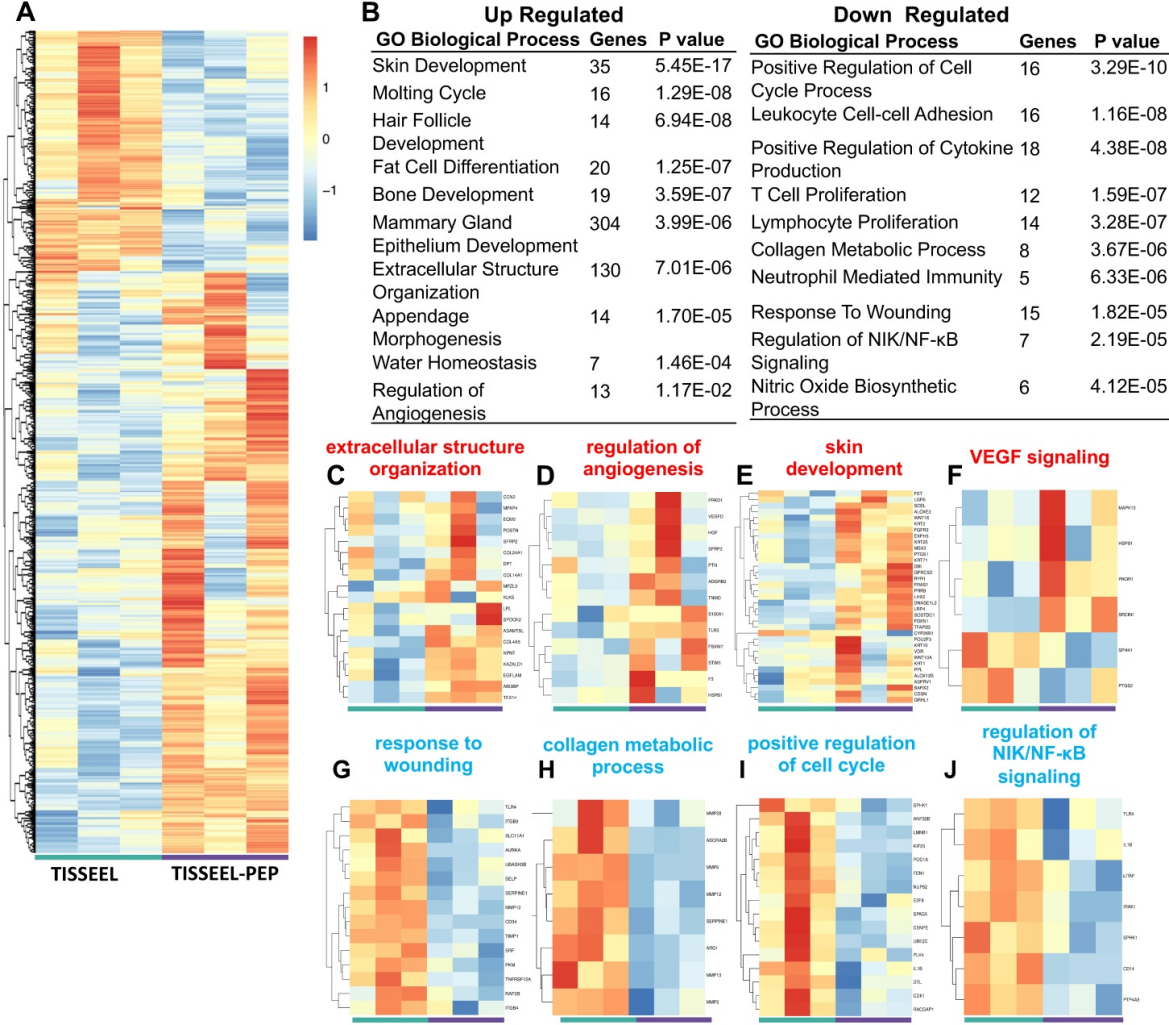
TISSEEL-PEP treatment mediated transcriptional changes of genes related to pro-wound healing events. (A): Differentially expressed genes in RNA-Seq of wound tissue from TISSEEL (left; green) and TISSEEL-PEP (right; purple) treated groups (N=3, genes were filtered with normalized counts of 100, filtered genes met the criteria of |log_2_FC|>0.5 and p<0.05 were considered significantly changed). (B): Gene ontology and pathway analysis of significantly differentially expressed genes. The top Upregulated and Downregulated 10 pathways were shown (p value less than 0.05). Most significant and nonredundant Biological Process or Pathways with respective gene number and p-value are shown. (C-J): Heatmaps of differentially regulated genes involved in the pro-wound healing processes including extracellular structure organization, regulation of angiogenesis, skin development, VEGF signaling, response to wounding, collagen metabolic process, positive regulation of cell cycle, regulation of NIK/NF-κB signaling.

Transcriptome profiling identified over 700 gene targets (Figure 6A) from tissue obtained at day 28 following treatment. The TISSEEL group served as the vehicle control in the analysis. 213 genes were significantly up-regulated, and 523 genes were down-regulated in the PEP-TISSEEL group. Gene ontology enrichment analysis and KEGG pathway analysis showed that sustained delivery of PEP modulated genes related to pro-wound healing (Figure 6B). Specifically, upregulation of gene expression related to extracellular organization, angiogenesis and skin development (Figure 6C-F). *In vivo* experiments not only validated the role of TISSEEL-PEP treatment in wound healing (Figure S10A), but also revealed intense CD31 and smooth muscle actin (SMA) staining are biomarkers of up-regulated VEGF expression (Figure S10B & C).

The downregulation genes participate in the wound effects, cell proliferation, collagen metabolism and NIK/NF-kB signaling (Figure 6G-J). Further analysis of downregulation events suggested the anti-inflammatory effects with TISSEEL-PEP treatment. These findings were further supported by PEP treatment stimulated M2-polarization of bone marrow derived macrophages, which promoted wound healing events through downregulation of the inflammatory responses (Figure S11A). Moreover, HE stained tissue sections of TISSEEL-PEP treated wounds demonstrated less inflammation events in the local area, as denoted by less overall immune cell infiltration (Figure S11B).

Overall transcriptome analysis pinpointed PEP effects with regulation of the TGF-β pathways. These findings are consistent with *in vitro* results that PEP regulates downstream mediators of TGF-β, including increased RHOA, SMAD2, TAK1 and RAS pathway (Figure 2), which promotes enhanced epithelization, fibroblast activation and collagen production. Interestingly, repressed collagen metabolism was observed in TISSEEL-PEP treated wounds, which suggests an earlier transition to the remodeling phase of wound healing 43,44. Transcriptional analysis and phenotypic evaluation of skin development and maturation revealed comparable findings.

## Discussion

Herein, we demonstrate the therapeutic potential of PEP biogel on ischemic wound healing. Despite storage at room temperature as a lyophilized powder, TGB-β containing PEP induced differentiation of epithelial cells and enhanced collagen deposition and organization at ischemic wound sites. Furthermore, TISSEEL-PEP treatment restored the normal skin architecture, gene expression and physiologic processes that favor wound healing of injured sites. The aforementioned benefits were not observed in the TISSEEL-only vesicles and control groups but was however weak in the PEP-only group. Ischemic wound healing is a complex process with overlapping phases. Rapid release like PEP-only group could only affect the early wound healing processes. However, TISEEL-PEP biogel sustains 5-10% PEP release for up to 2 weeks, which regulate latter stages of wound healing (Figure 6C-F). The concert of biological events driven by TISSEEL-PEP resulted in regenerated tissue that had properties akin to normal skin in histological, biomechanical and functional assessment. Global transcriptome fingerprint of PEP-devoid versus PEP-potentiated treatment groups highlighted normalization of molecular events towards a state of health. Taken together, the data presented herein demonstrate that our findings further demonstrate that PEP based exosomes are stable at room-temperature, with the encapsulated TGF-β promoting ischemic wound healing. PEP donation of TGF-β into the wound microenvironment likely serves as the initial trigger for acceleration of wound healing, as is suggested by loss of impact following TGF-β neutralization. However, perseverance of TGF-β signaling noted in this manuscript is unlikely to be from PEP four weeks after treatment, but rather driven by the host's own tissue microenvironment in the later stages of wound healing. As a lyophilized exosome product manufactured under current good manufacturing practices, application of this technology to a rabbit ischemic wound model has provided the necessary justification to secure an Investigational New Drug application for use of PEP in man (19567).

Ischemic wounds are the underlying cause for significant morbidity and mortality worldwide. As treatment options are limited, regenerative approaches have been considered to address this unmet need 45. The rabbit ear ischemia model was here utilized to enhance the translational impact of this work as well as to provide a model that preserves skin continuity and lacks the capacity for spontaneous revascularization 46. This work hereby serves as a proof of concept for the use of cell-free exosome-based therapies to induce regenerative events in refractory wounds. Cell-based approaches are encumbered by scalability, logistical issues and cost 47,48. In contrast, cell-independent platforms, such as exosome therapies provide the opportunity to deliver a uniform biologic without concern for cell survival or altered behavior in response to divergent product handling or host microenvironments 49,50. This study implemented the use of an exosome technology combined with a clinical-grade surgical biogel to accelerate ischemic wound healing through the promotion of angiogenesis, collagen reorganization and skin regeneration. The data presented herein shows that inert biogels alone could not achieve regenerative benefit in comparison to biogels following exosome biopotentiation. PEP, stored in lyophilized form, is a cell-independent regenerative exosome with high purity and uniformity capable of preserving growth factor bioactivity despite remaining at room-temperature.

Indeed, the establishment of this off-the-shelf approach to regenerative wound care augments clinical ease of use and fosters broad dissemination of this technology for implementation as a point of care wound therapeutic platform, devoid of the infrastructural needs typically required with cell-based therapies. Elimination of the multi-step process needed for freeze and thaw biologics-based therapies not only facilitates ease of use to enhance therapeutic equipoise as therapeutic doses delivered to target populations.

Evidence of positive impact on multiple biological activities in healing process was observed in this study. Angiogenic events were observed on histology in the TISSEEL-PEP group, suggesting that PEP may also target endothelial cell activity. This activity was tracked through evaluation of endothelial tube formation in a co-culture with human dermal fibroblasts as previously described 51. This was furthermore corroborated *in vivo* as transcriptome profiling revealed higher expression of genes, including VEGF signaling, related to angiogenesis. Furthermore, tissue transcriptome profiling suggested downregulation of inflammatory and NIK/NF-κB related events suggesting that indeed PEP likely has a polyvalent action in tissues to drive regenerative events.

Our study shows that PEP biogel normalized chronic skin structure and reepithelization, promoting rapid wound healing. Although PEP-mediated impact on skin regeneration was here shown to be driven by a TGF-β centric program, transcriptome profiling of regenerated tissue suggests a polyvalent mode of action from this exosome to also drive angiogenic and immunomodulatory events. Furthermore, its application here was in an animal model system, clinical translation will be required to identify an optimal course of treatment and dosing in patients with chronic wounds.

## Conclusion

In summary, our study showed a specialized PEP biopotentiated hydrogel facilitated rabbit ischemic wound healing through regulating epithelial transdifferentiation, collagen reorganization and overall guiding skin tissue development via the TGF-β pathway. Our findings offer a cell-free regenerative therapy with promising therapeutic potential for patients with chronic ischemic wounds. As the findings of this study provide the foundational basis for a new clinical trial, this technology can now serve as a prototype for the implementation of off-the-shelf cell-independent regenerative therapies in man.

## Supplementary Material

Supplementary figures and tables.

Supplementary table S2.

Supplementary movie S1.

## Figures and Tables

**Table 1:** PCR primer sequences

	Forward	Reverse
GADPH	GAGTCAACGGATTTGGTCGT	TTGATTTTGGAGGGATCTCG
H-Ras	ACGACGATGACAAGA CGGAA	ATGGCGCTGTACTCCTCCT
Smad2	ACTAACTTCCCAGCAGGAAT	GTTGGTCACTTGTTTCTCCA
Erk1	CCTGCGACCTTAAGATTTGTGATT	CAGGGAAGATGGGCCGGTTAGAGA
TIMP-1	TGACATCCGGTTCGTCTACA	TGCAGTTTTCCAGCAATGAG
Periostin	ATGATTCCCTTTTTACCCATGTTTTCTCTA	GAAGGAATAATCATGCCATTTTTTAAGTCC
MKK3	CTTGGTGACCATCTCAGAACTGG	CTTCTGCTCCTGTGAGTTCACG
P38	CCAATGCCTACGACAAGACAGC	TGGGAAGTGACCTCGTTTGCCA
Nf-kb	GCAGCACTACTTCTTGACCACC	TCTGCTCCTGAGCATTGACGTC
hRHOA	CGCTTTTGGGTACATGGAGT	TTGCAGCAAGGTTTCACAAG
Akt	TGGACTACCTGCACTCGGAGAA	GTGCCGCAAAAGGTCTTCATGG
TAK1	CAGAGCAACTCTGCCACCAGTA	CATTTGTGGCAGGAACTTGCTCC
NOS2	GTG GCA GGA CAT GAA GAA GAA	CAT CAG CAC AGA GGC AAA GA
TNF-a	CTC ATC TAC TCC CAG GTT CTC T	GTT GAC CTT GTT CGG GTA GG
IL-6	GTC AAC TGC ATG AAC AGA AAG G	AGC AGG CAG GTC TCA TTA TTC
IL-1b	CGA ACC CAA GCT ACA GGA ATA G	TGG AAA GTG TGT GTC CAA TCA
IL-10	CCT GTG GGA TTT GAG TGT CTT A	GCT CGG CTT AGG AGT TAG AAA G
CD206	GGT GAC ATC CAC GAC TAC TTT AG	CCA GGC ATA GCT GTT GTA CTT

## Abbreviations

Alix: ALG-2-interacting protein X
cDNA: complementary DNA
COL1A: collagen type I
COL3A: collagen type III
3D-EM: three-dimensional electron microscopy
ELISA: enzyme-linked immunosorbent assay
ERK1: extracellular signal-regulated kinases 1
EVs: extracellular vesicles
GAPDH: glyceraldehyde-3-phosphate dehydrogenase
GFP: green fluorescent protein
hFB: human fibroblasts
hKC: human keratinocytes
HUVECs: human umbilical vascular endothelial cells
MKK3: mitogen-activated protein kinase kinase 3
PEP: purified exosome product
qRT-PCR: quantitative real-time polymerase chain reaction
rFB: rabbit dermal fibroblasts
RHOA: ras homolog family member A
TAK1: transforming growth factor beta-activated kinase 1
TEM: transmission electron microscope
TGF-β: transforming growth factor beta
VEGF: vascular endothelial growth factor

## Supporting Information

Representative transmission electron microscopic image of PEP exosomes from each batch; protein Concentration from different batches; releasing criteria data from different batches; schematic of TISSEEL-PEP preparation and representative scanning electron microscopic image of PEP incorporated biogel; gross picture of each individual animal group up to a 28-day follow-up. quantification of the healing area up to 28 days of follow-up; H&E staining of wound bed from each individual animal; angiogenesis of wound bed from each individual animal; inflammation of wound bed from each individual animal; masson trichrome staining of each individual animal; TISSEEL-PEP (20%) release assessment; PCR primer sequences; 3D-EM movie of different tissue groups.
