# Supplementary material for: TGF-β loaded exosome enhances ischemic wound healing in vitro and in vivo
Source: Theranostics. 2021 Apr 30;11(13):6616–31. doi: 10.7150/thno.57701 (PMC8120220; doi:10.7150/thno.57701)
Supplement: Supplementary file 1 — Supplementary figures and tables. [file thnov11p6616s1.pdf]

Supplementary materials

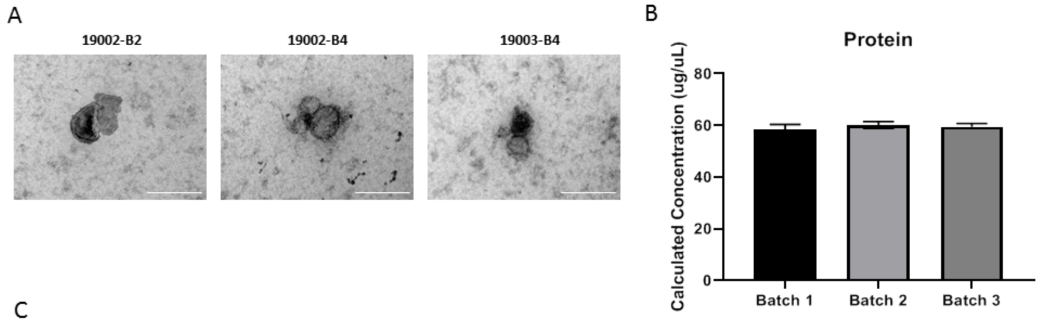

C

Batch Number	Moisture (%)	Sterility	Endotoxin EU/mL	Cell proliferation rate (%)
19002-B2	6.33	No growth	<0.100	99.89± 0.047
19002-B4	5.86	No growth	<0.100	99.48± 0.132
19003-B4	6.09	No growth	<0.100	99.74± 0.113

Figure S1. PEP batches have consistent features. (A): Representative transmission electron microscopic image of PEP exosomes from each batch. Scale bar, 200 nm. (B): Protein Concentration from different batches. (C): Stability test data from different batches.

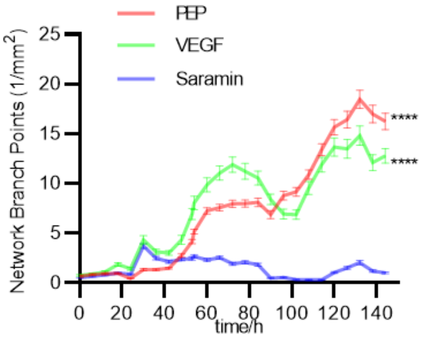

Figure S2. PEP promoted HUVECs angiogenesis in vitro. Quantification of branch points was performed every 6 hours over a 6 day period. ****$p < 0.0001$.

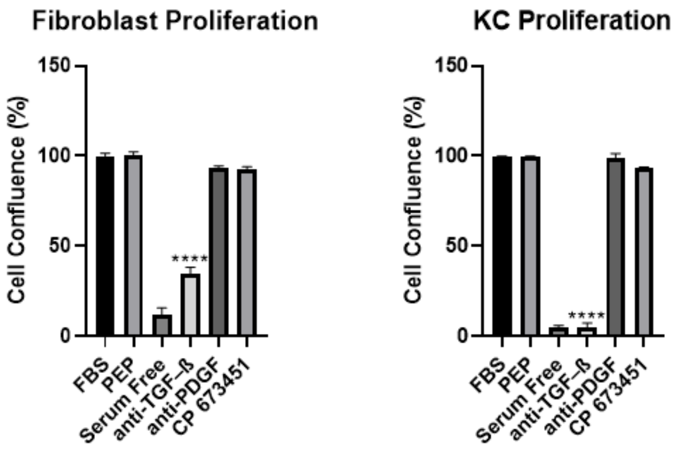

Figure S3. PEP promoted fibroblast and keratinocyte proliferation in vitro though exosomal TGF-β. Quantification of cell confluence was performed. ****$p < 0.0001$.

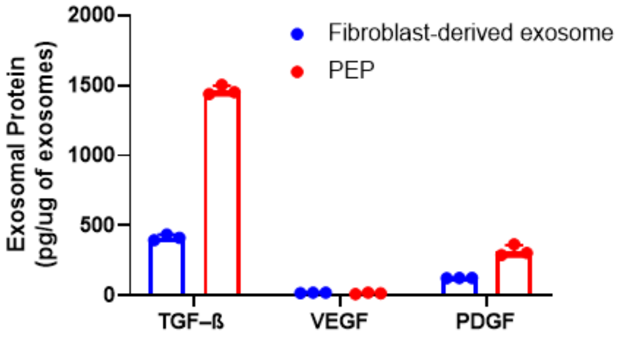

Figure S4. Growth factor evaluation of PEP and fibroblast-derived exosomes**.**

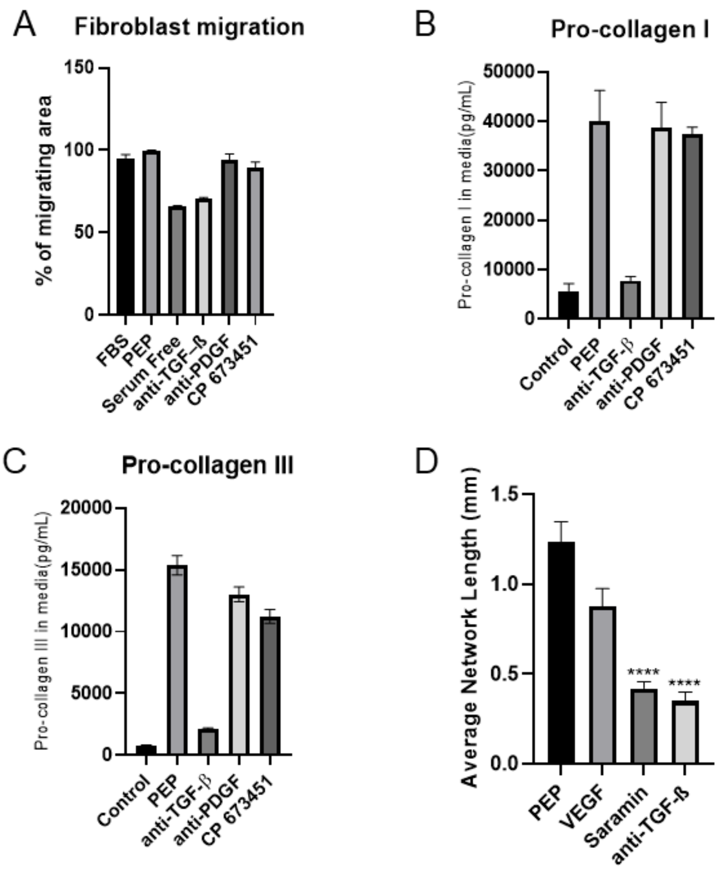

Figure S5. Exosomal TGF-β inhibition suppressed PEP induced cellular events, including cell migration(A), collagen synthesis (B,C), and vascular tube formation (D).

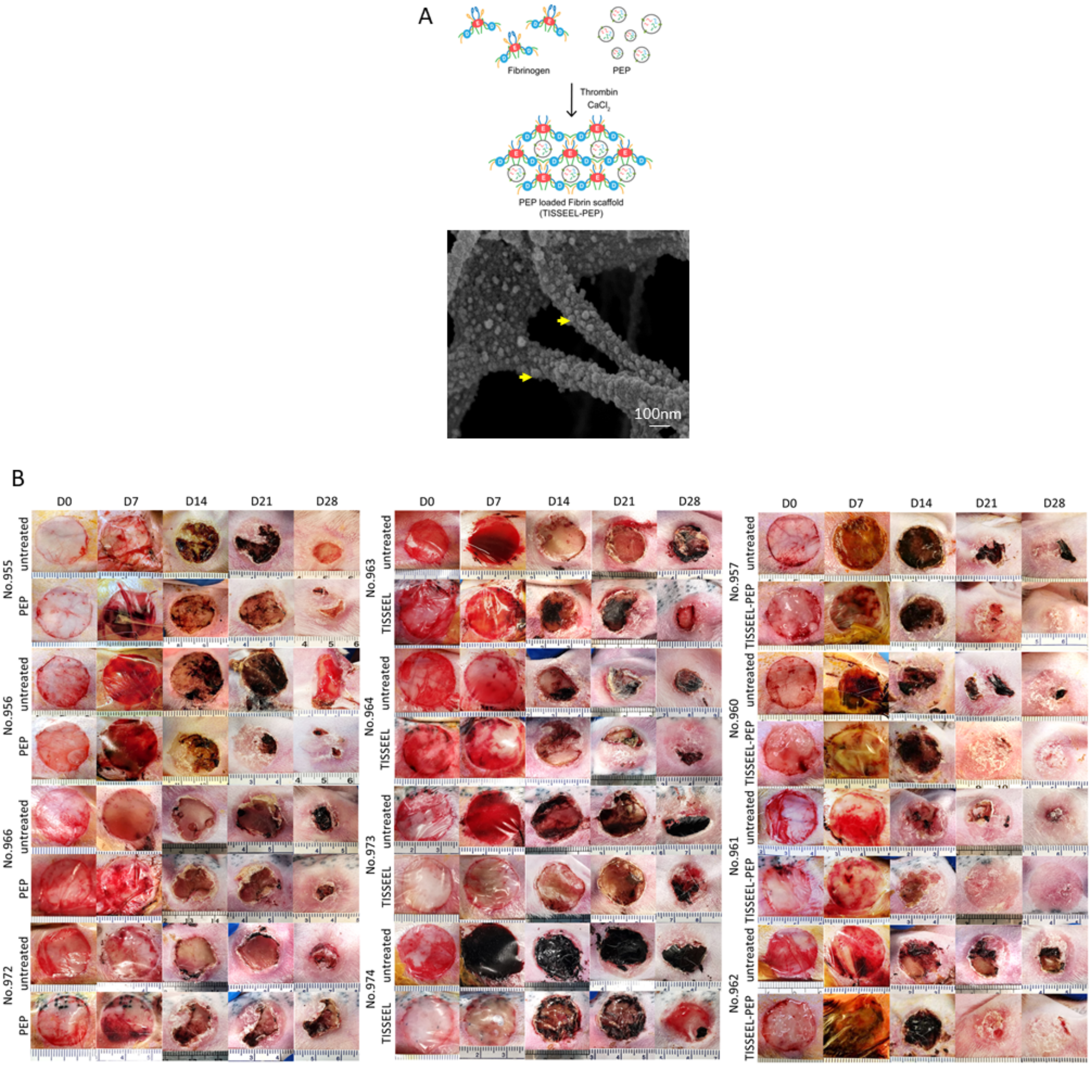

Figure S6. TISSEEL-PEP biogel drives in vivo wound healing (A): Schematic of TISSEEL-PEP preparation and representative scanning electron microscopic image of PEP incorporated biogel. Yellow arrow: PEP. (B): Gross picture of each individual animal group up to a 28-day follow-up. Of note, in animals with PEP exposure, contralateral ears were noted to heal better versus those without any PEP exposure (Untreated/TISSEEL grp)

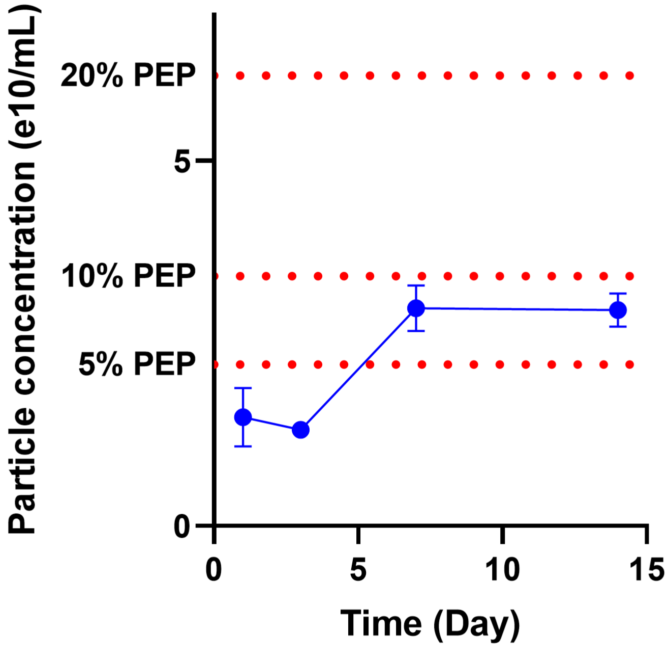

Figure S7. TISSEEL-PEP(20%) release assessment. The PEP release profile of TISSEEL-PEP biogel was tested on day 1, 3, 7 and 14 with exosome release normalized to 5%, 10% and 20% PEP standards (y-axis and dotted lines). PEP concentration in TISSEEL was adjusted to achieve release concentrations equivalent to 5% of PEP in solution.

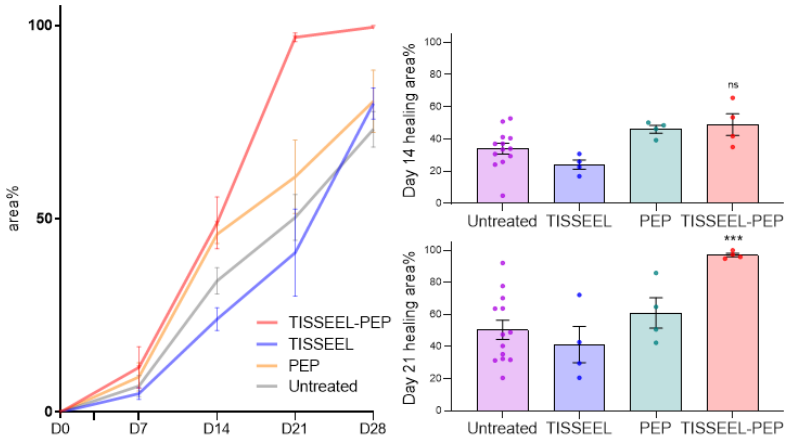

Figure S8. Quantification of the healing area up to 28 days of follow-up. 2-tailed unpaired Student's t test for each group compared with untreated control group. *p < 0.05, **p < 0.01, ***p < 0.001.

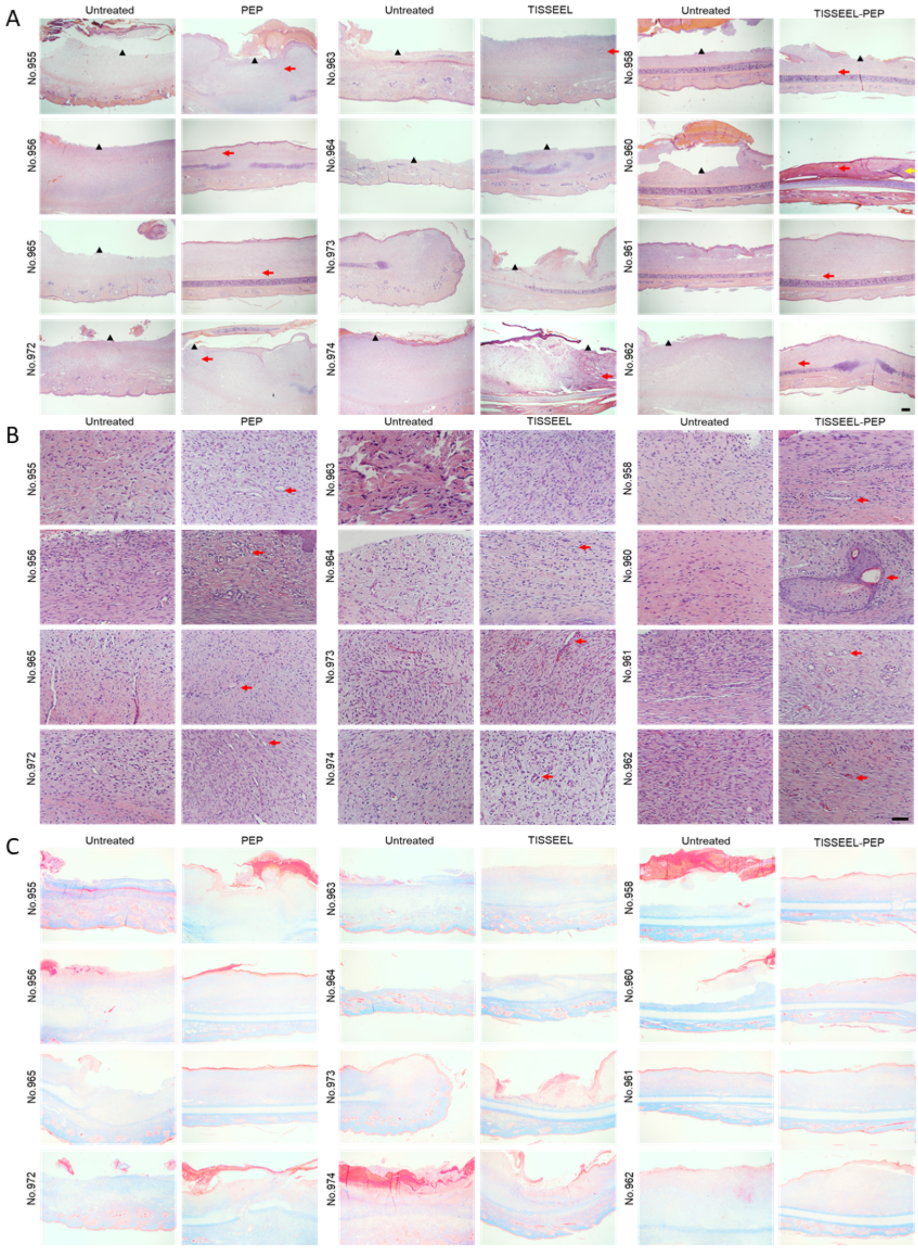

Figure S9. Histology assessment of each individual animal. (A): H&E staining of wound bed from each individual animal. Red arrow: new blood vessel. (B): Angiogenesis of wound bed from each individual animal. Red arrow: new blood vessel. (C): Masson trichrome staining of each individual animal. Scale bar in Figure A&C: 100 µm. Scale bar in Figure B: 50 µm.

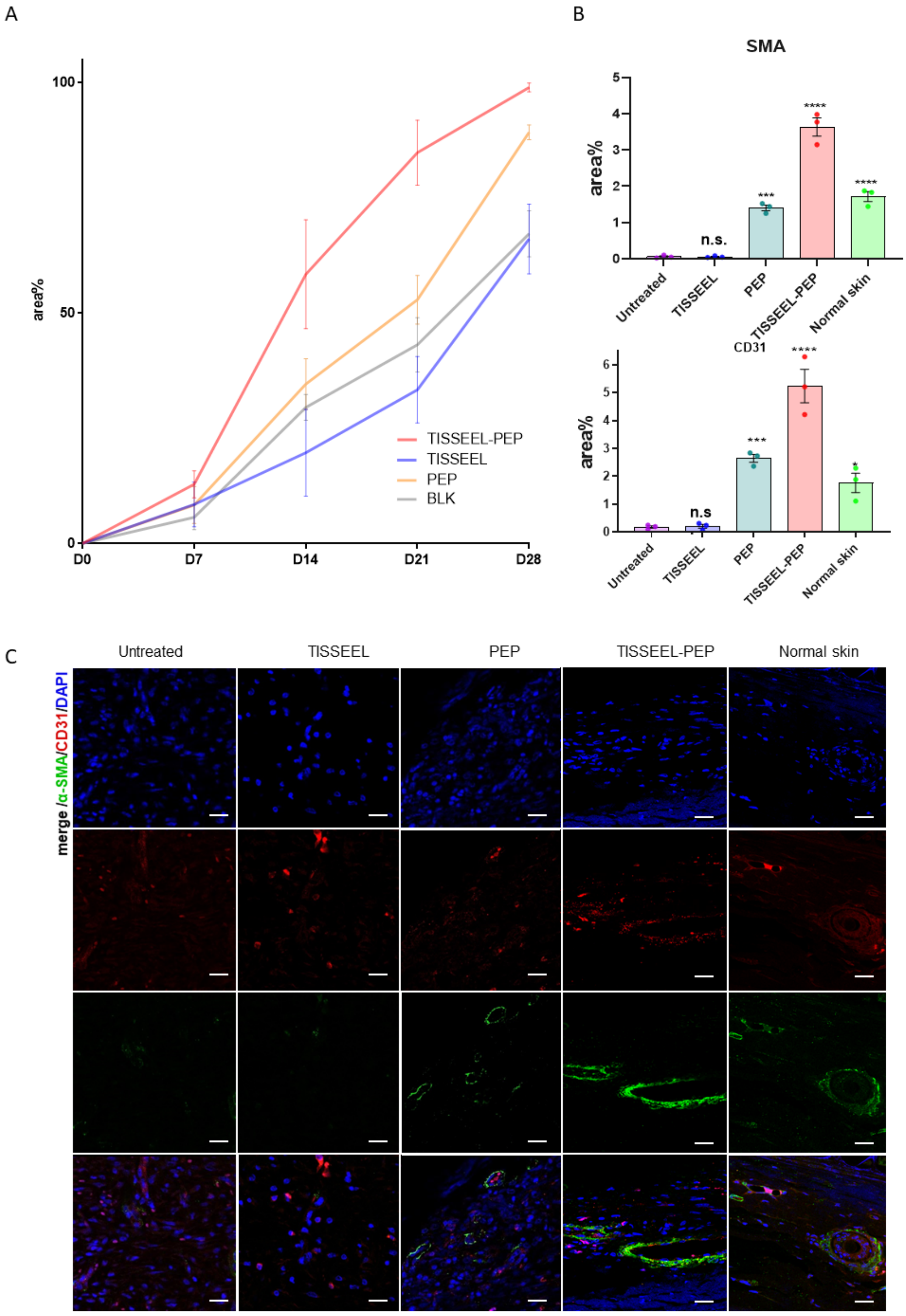

Figure S10. Additional in vivo studies. (A): Quantification of the healing area up to 28 days of follow-up. (B&C): CD31 and SMA immunofluorescent staining of wounds from different groups. Scale bar in Figure C: 20 μm. *p < 0.05, ***p < 0.001, ****p < 0.0001, n.s.= non-significant.

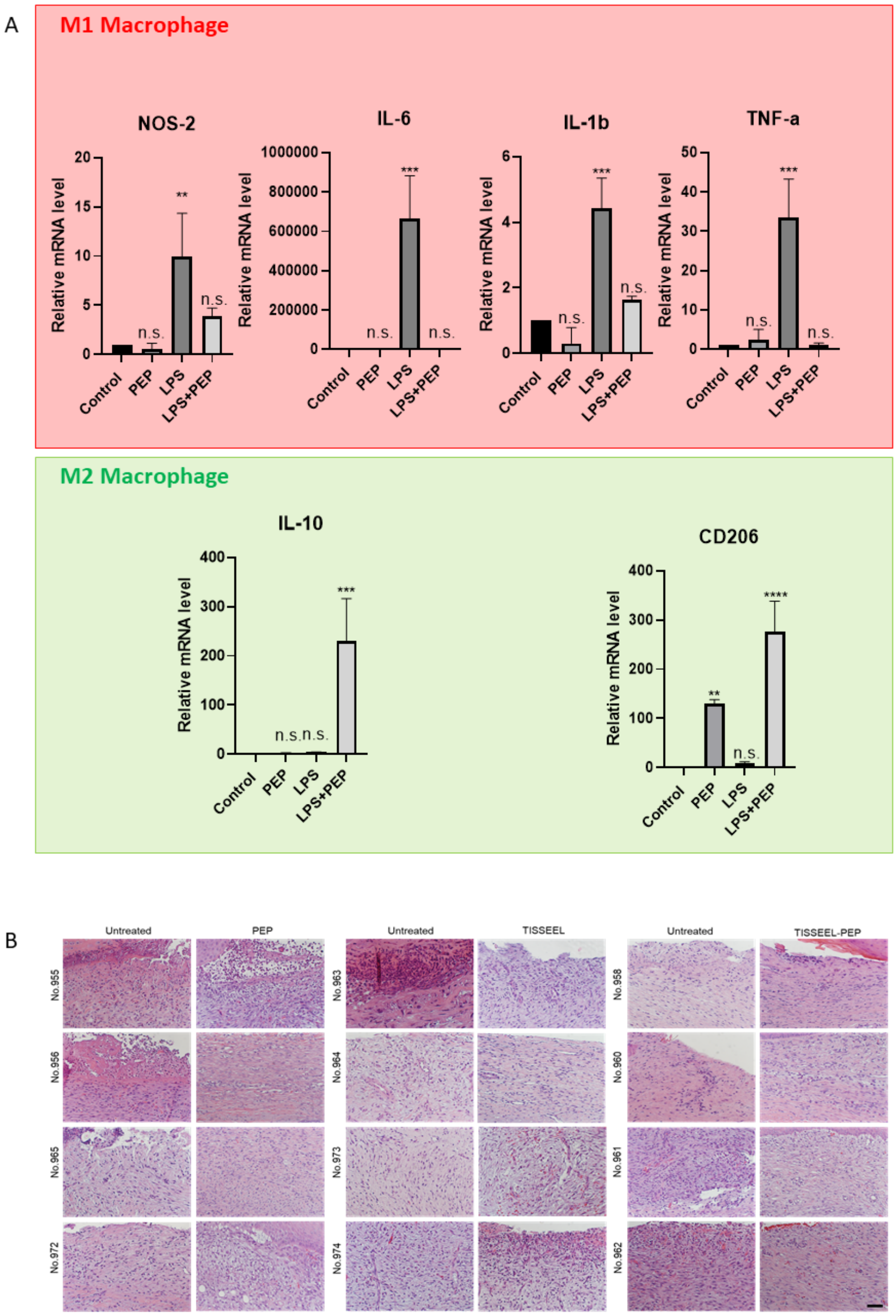

Figure S11. PEP stimulated anti-inflammatory response both *in vitro* and *in vivo*. (A): PEP induced macrophage M2 polarization upon LPS stimulation. qRT-PCR analysis of M1(Red) and M2 (Green) signature gene expression displayed as the fold change compared with the normal macrophage control at 24 hours post treatment. (B): Inflammation evaluation of wound bed from each individual animal. Scale bar in Figure B: 50 μm. **p < 0.01, ***p < 0.001, ****p < 0.0001, n.s.= non-significant.

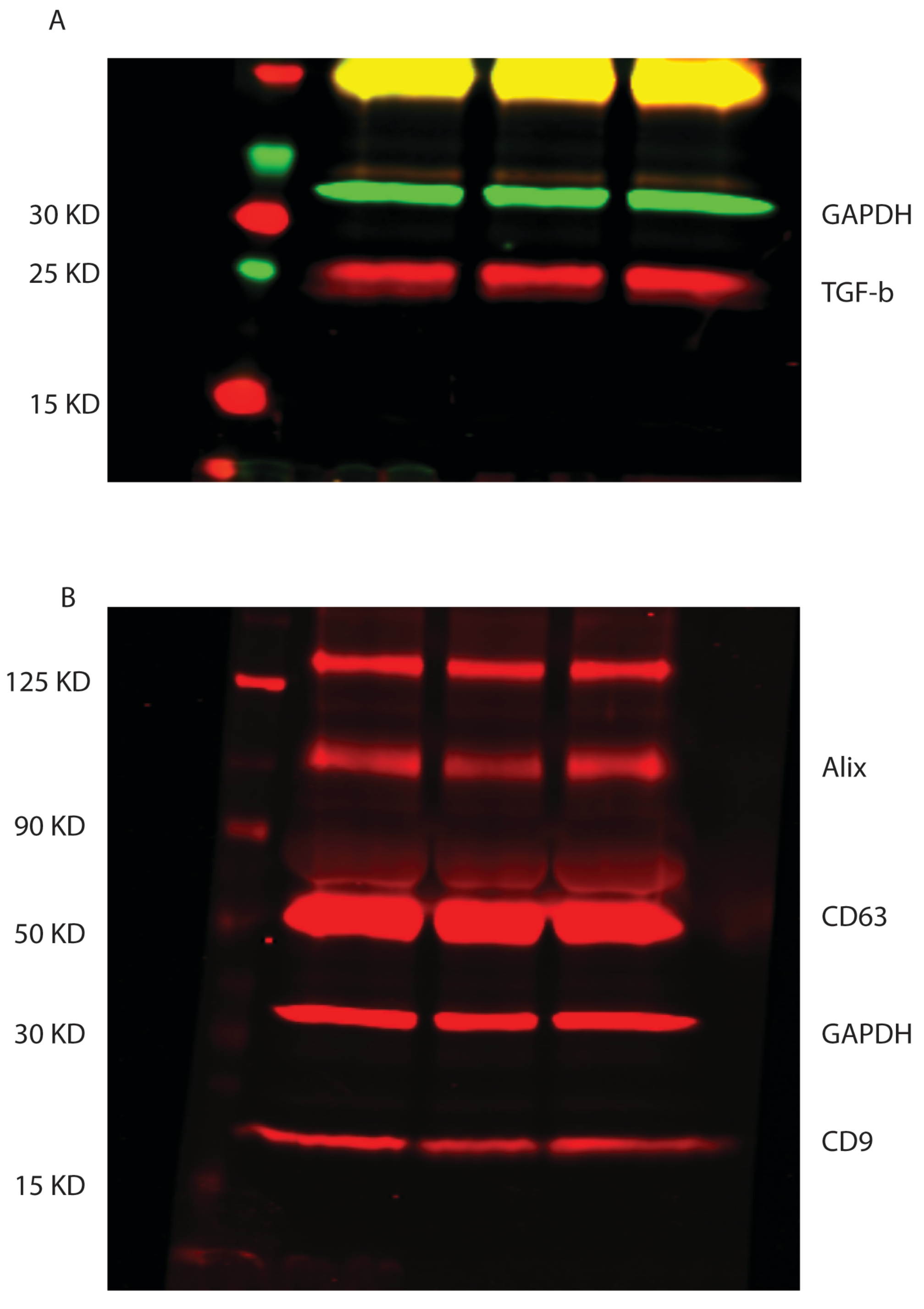

Figure S11. Whole blots of western blot analysis.

Table 1. PCR primer sequences

	Forward	Reverse
GAPDH	GAGTCAACGGATTTGGTCGT	TTGATTTTGGAGGGATCTCG
H-Ras	ACGACGATGACAAGA CGGAA	ATGGCGCTGTACTCCTCCT
Smad2	ACTAACTTCCCAGCAGGAAT	GTTGGTCACTTGTTTCTCCA
Erk1	CCTGCGACCTTAAGATTTGTGATT	CAGGGAAGATGGGCCGGTTAGAGA
TIMP-1	TGACATCCGGTTCGTCTACA	TGCAGTTTTCCAGCAATGAG
Periostin	ATGATTCCCTTTTTACCCATGTTTTCTCTA	GAAGGAATAATCATGCCATTTTTAAGT[illegible]
MKK3	CTTGGTGACCATCTCAGAACTGG	CTTCTGCTCCTGTGAGTTCACG
P38	CCAATGCCTACGACAAGACAGC	TGGGAAGTGACCTCGTTTGCCA
Nf-kb	GCAGCACTACTTCTTGACCACC	TCTGCTCCTGAGCATTGACGTC
hRHOA	CGCTTTTGGGTACATGGAGT	TTGCAGCAAGGTTTCACAAG
Akt	TGGACTACCTGCACTCGGAGAA	GTGCCGCAAAAGGTCTTCATGG
TAK1	CAGAGCAACTCTGCCACCAGTA	CATTTGTGGCAGGAACTTGCTCC
NOS2	GTG GCA GGA CAT GAA GAA GAA	CAT CAG CAC AGA GGC AAA GA
TNF-a	CTC ATC TAC TCC CAG GTT CTC T	GTT GAC CTT GTT CGG GTA GG
IL-6	GTC AAC TGC ATG AAC AGA AAG G	AGC AGG CAG GTC TCA TTA TTC
IL-1 b	CGA ACC CAA GCT ACA GGA ATA G	TGG AAA GTG TGT GTC CAA TCA
IL-10	CCT GTG GGA TTT GAG TGT CTT A	GCT CGG CTT AGG AGT TAG AAA G
CD206	GGT GAC ATC CAC GAC TAC TTT AG	CCA GGC ATA GCT GTT GTA CTT

Table 2. Full gene list of transcriptome change (Attached)
